# The Relationship and Gender Disparity Between Thyroid Nodules and Metabolic Syndrome Components Based on a Recent Nationwide Cross-Sectional Study and Meta-Analysis

**DOI:** 10.3389/fendo.2021.736972

**Published:** 2021-09-21

**Authors:** Fan Zhang, Yongze Li, Xiaohui Yu, Xichang Wang, Zheyu Lin, Bo Song, Lijun Tian, Chuyao Feng, Zhongyan Shan, Weiping Teng

**Affiliations:** Department of Endocrinology and Metabolism, Institute of Endocrinology, National Health Commission Key Laboratory of Diagnosis and Treatment of Thyroid Diseases, The First Affiliated Hospital of China Medical University, Shenyang, China

**Keywords:** gender, metabolic syndrome, meta-analysis, thyroid nodule, TIDE

## Abstract

**Background:**

Metabolic syndrome (MetS) has a potential connection with thyroid disease, but its relationship with thyroid nodules (TNs) is still controversial. This study aims to clarify the relationship between MetS and TNs, and this relationship in the subgroup of gender.

**Methods:**

The recent nationwide cross-sectional study called Thyroid Disorders, Iodine Status, and Diabetes Epidemiological survey provided the newest data on the relationship between MetS and TNs from China and included 56,729 subjects. We also researched related literature in PubMed, EMBASE, Cochrane Library, and MEDLINE until Oct 30, 2020, in order to perform a meta-analysis. The relevant articles were examined, and the eligible studies were included to assess the association between MetS and TNs.

**Results:**

The meta-analysis included 15 studies (involving 468,845 subjects). Of these, 14 studies were from the databases, and one study was this cross-sectional data. The meta-analysis showed that TNs were associated with a higher prevalence of MetS (OR=1.87, 95% CI: 1.44–2.45) and the components of MetS, including central obesity (OR=1.41, 95% CI: 1.15–1.72), hypertriglyceridemia (OR=1.13, 95% CI: 1.10–1.15), low high-density lipoprotein cholesterolemia (OR=1.11, 95% CI: 1.02–1.20), abnormal blood pressure (OR=1.68, 95% CI: 1.62–1.75), and hyperglycemia (OR=1.59, 95% CI: 1.46–1.74). Central obesity displayed gender differences, being a risk factor in males (OR=1.38, 95% CI: 1.02–1.86) but not in females (OR=1.47, 95% CI: 0.97–2.23).

**Conclusion:**

TNs were indeed associated with a higher prevalence of MetS. In addition, its component diseases, such as central obesity, hypertriglyceridemia, abnormal blood pressure, and hyperglycemia, were also associated with TNs. Females with MetS or its components had a higher risk of suffering from TNs than males.

## Introduction

Metabolic syndrome (MetS) is a clinical condition characterized by a series of metabolic risk factors. According to the International Diabetes Federation, the American Heart Association, and the National Heart, Lung, and Blood Institute, MetS is defined as the simultaneous occurrence of at least three of the following components: central obesity, abnormal glucose metabolism, increased blood pressure (BP), dyslipidemia, and low high-density lipoprotein cholesterol (HDL-C) (1). Several previous studies have reported that impaired glucose metabolism is an independent risk factor for increased thyroid volume and increased prevalence of nodules (2–4). Obesity is associated with higher risks of thyroid nodules (TN) and thyroid cancer (5–7). Insulin resistance (IR) was also shown to promote the formation and growth of TNs (8). Recently, it has been suggested that MetS is associated with the functional and morphological alterations of the thyroid gland and may be involved in the pathogenesis of TNs (9, 10).

TNs are defined as discrete lesions within the thyroid gland that are radiologically distinct from the surrounding thyroid parenchyma (11). An estimated 16 million individuals in the US have a palpable nodule, and, due to the rapid development of imaging technology, up to 219 million individuals have an ultrasound-detectable nodule (12). In the TIDE study in China, the prevalence of TNs is over 20% and shows a significant difference between genders. These TNs include several subgroups, such as harboring significant cancer (approximately 10%) and huge lumps causing (or is the risk of) compressive symptoms (5%) and thyroid dysfunction (5%). About 90% of TNs are benign, while 95% are asymptomatic and remain so during follow-up. Asymptomatic and benign nodules can be managed safely with a less intensive follow-up protocol to find malignant change in time (12). Similar to MetS, several studies have found a significant gender difference in the prevalence of TNs (13, 14).

A few studies in recent years have reported a relationship between MetS and TNs, but the conclusions have not been consistent. In this study, we used the results of the TIDE data and reviewed all related pieces of literature on the website to conduct this meta-analysis, the aim of which was to estimate the relationship between TNs and MetS. In addition, a subgroup analysis contained gender and the components of MetS.

## Materials and Methods

### TN and MetS Ratio in the TIDE Study

The TIDE survey is a national epidemiological cross-sectional study that was conducted from 2015 to 2017. The results of the relevant studies have been reported before (15–17). The personal information and blood sample collection process and standards of all subjects refer to our previous research results (17). In this study, the inclusion criteria for the subjects were as follows: aged 18 years or older; no use of iodine drugs or contrast within three months; not pregnant. The exclusion criteria were as follows: major data missing; abnormal levels of thyroid function; history of thyroid disease; other chronic wasting diseases.

Ultrasonography of the thyroid was performed by specially trained technicians using a GE Logiq a100 with a 7.5-MHz linear array transducer. A TN was defined as a discrete lesion within the thyroid gland that was radiologically distinct from the surrounding thyroid parenchyma.

The diagnosis criteria of MetS were defined as more than three of the following criteria being apparent in a participant:

(1) Central obesity: waist circumference: male ≥90 cm, women ≥85 cm;

(2) Hypertriglyceridemia: TG ≥1.7 mmol/L;

(3) Low HDL-C: HDL-C level: males <1.0 mmol/L, females, <1.3 mmol/L;

(4) Abnormal BP: the presence of any of the following three, criteria: (a) taking antihypertensive medications; (b) systolic, BP (SBP) ≥130 mmHg; (c) diastolic BP (DBP) ≥85 mmHg;

(5) Hyperglycemia: any of the following four criteria related, to blood glucose: (a) self-reported diabetes history or taking, hypoglycemic drugs; (b) fasting plasma glucose (FPG) ≥5.6, mmol/L; (c) OGTT-2hPG ≥7.8 mmol/L; (d) HBA1c ≥5.7%.

### Other Literature From Websites: Search Strategy and Selection Criteria

A systematic literature search (PubMed, EMBASE, Cochrane Library, and MEDLINE) was performed from inception to Oct 2020. The keywords “thyroid nodule” and “metabolic syndrome” were used to search for potentially relevant studies in the databases mentioned above. The reference lists of the retrieved articles were scanned to identify additional studies and expand our search.

#### Inclusion and Exclusion Criteria

**Figure 1:**
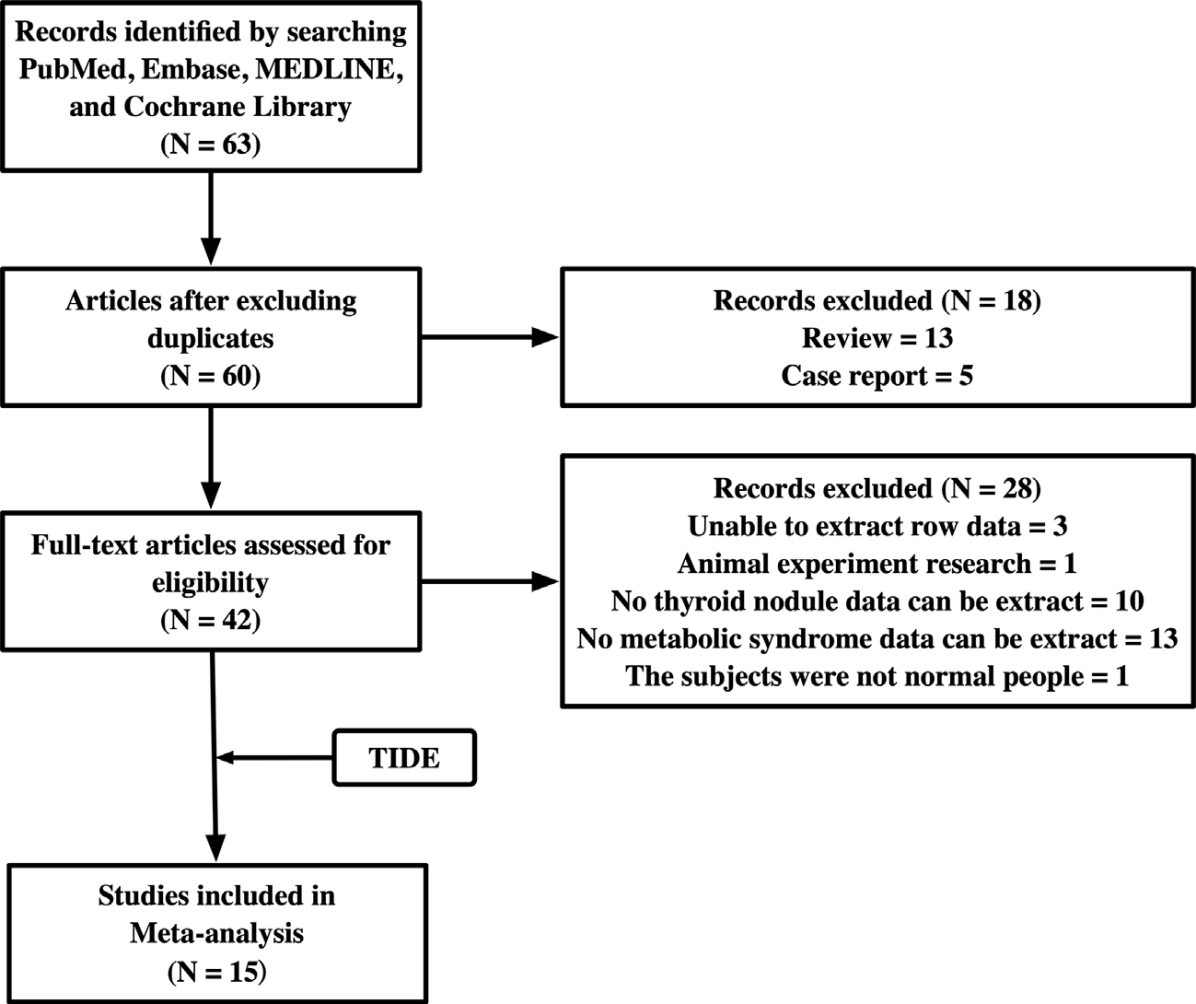
Flow chart of the study selection process.

Studies were considered eligible if they met the following criteria: investigated the relationship between TNs and MetS; data on TNs and MetS could be extracted to analyze the 95% confidence interval (CI). Comments, conference abstracts, books, reports, reviews, and articles that could not be analyzed further were excluded. We also excluded two studies, one in which the subjects had colon polyps, and the other was an animal experiment. The flowchart of the studies included is shown in **Figure 1**.

#### Data Extraction

Two authors carefully extracted the following information from each study: first author’s name, publication year, race, country of population, and diagnostic criteria for MetS. In addition, we collected primary data on TNs and MetS.

#### Quality Assessment of the Included Studies

We used the Newcastle-Ottawa scale (NOS) to assess the quality of the selected studies from three aspects: selection, comparability, and results, with a maximum of four, two, and three stars, respectively. Studies assigned >6 stars are considered high-quality studies (18).

### Statistical Analysis

SPSS 26.0 software (Chicago, IL, USA) was used to statistically review the number of subjects with TNs and MetS with its constituent diseases from the TIDE study. Categorical data presented as counts and percentages were analyzed using the chi-square and Fisher’s exact tests. Adjusted odds ratios (ORs) with 95% CI were calculated by multivariable logistic regression to examine the association between MetS, its components, and the prevalence of TNs.

The meta-analysis was performed using Revman Manager 5.4 (Cochrane Collaboration, Oxford, UK). The OR was used to compare dichotomous variables. All results were estimated with 95% CIs. A p-value <0.05 was considered statistically significant. Heterogeneity was examined by using the Q-test and I^2^ statistic. A fixed-effect model was used when p > 0.1 and I^2^ < 50%; otherwise, a random-effects model was applied. Possible publication bias was tested using Begg’s funnel plot.

## Results

### The Ratio of TNs and MetS With Its Component Diseases in the TIDE Study

**Table 1** shows the ratio of TNs and MetS and their relationship as found in the TIDE study, which included 56,729 participants. The prevalence of MetS is 28.7% in the entire population of China, in which there are more males (32.9%) than females (24.2%). The prevalence of MetS in the population with TNs (38.2%) is significantly higher than in the population without TNs (26.6%, p < 0.001) regardless of gender. As for the component diseases of MetS, the prevalences of all are over one-fifth, and the abnormal BP (43.8%) and hyperglycemia (45.8%) reach nearly half. In addition, regarding TN status, MetS and its component diseases display a significant difference between the groups with and without TNs, except low HDL-C. The prevalence of metabolic disorders is higher in the group with TNs than without. Furthermore, the prevalence of metabolic disorders (except low HDL-C) is higher in males than females regardless of TN status. As for the low HDL-C, there is a difference between the TN group and the non-TN group (p = 0.025). However, in considering gender differences, there is no significant difference between the TN group and the non-TN group (males, 0.848; females, 0.564). In addition, the prevalence in males is significantly lower than in females.

**Table 1:** Associations between metabolic syndrome and thyroid nodules in TIDE survey.

Parameters, % (n)	Overall	Non-Thyroid Nodule	Thyroid Nodules	*p*	OR	95%CI	*p* value
	(N = 56729)	(N = 46099)	(N = 10630)				
MetS	28.7 (16308)	26.6 (12251)	38.2 (4057)	*0.000*	1.069	1.011-1.129	0.018
Male	32.9 (9733)	31.2 (7769)	42.3 (1964)	*0.000*	1.132	1.047-1.224	0.002
Female	24.2 (6575)	21.2 (4482)	35.0 (2093)	*0.000*	0.999	0.923-1.081	0.985
Central obesity	34.4 (19526)	32.2 (14864)	43.9 (4662)	*0.000*	1.108	1.044-1.175	0.001
Male	39.0 (11518)	37.3 (9303)	47.7 (2215)	*0.000*	1.120	1.027-1.220	0.010
Female	29.5 (8008)	26.3 (5561)	40.9 (2447)	*0.000*	1.081	0.996-1.174	0.063
Hypertriglyceridemia	28.2 (15977)	27.6 (12741)	30.4 (3236)	*0.000*	0.969	0.920-1.020	0.229
Male	34.8 (10285)	34.5 (8600)	36.3 (1685)	*0.018*	0.980	0.912-1.053	0.575
Female	21.0 (5692)	19.6 (4141)	25.9 (1551)	*0.000*	0.950	0.882-1.023	0.175
Low HDL-C	20.3 (11492)	20.1 (9255)	21.0 (2237)	*0.025*	0.899	0.849-0.951	0.000
Male	14.7 (4333)	14.7 (3657)	14.6 (676)	*0.848*	0.954	0.868-1.048	0.326
Female	26.4 (7159)	26.4 (5598)	26.1 (1561)	*0.564*	0.869	0.810-0.933	0.000
Abnormal BP	43.8 (24835)	41.3 (19051)	54.4 (5784)	*0.000*	1.129	1.073-1.188	0.000
Male	52.2 (15444)	50.4 (12564)	62.0 (2880)	*0.000*	1.080	1.004-1.161	0.038
Female	34.6 (9391)	30.6 (6487)	48.5 (2904)	*0.000*	1.170	1.090-1.256	0.000
Hyperglycemia	45.8 (26008)	43.3 (19978)	56.7 (6030)	*0.000*	1.070	1.019-1.123	0.007
Male	48.5 (14348)	46.5 (11593)	59.3 (2755)	*0.000*	1.079	1.005-1.159	0.036
Female	42.9 (11660)	39.6 (8385)	54.7 (3275)	*0.000*	1.063	0.994-1.137	0.075

TIDE, Thyroid disorders, Iodine status and Diabetes Epidemiological; MetS, metabolic syndrome; HDL-C, high density lipoprotein cholesterol; BP, blood pressure.

Logistic regression results show that several MetS indicators, except for hypertriglyceridemia (OR=0.969, 95%CI: 0.920-1.020, p = 0.229), are associated with TNs. For gender differences, MetS, central obesity, abnormal BP, and hyperglycemia are risk factors for TNs in males (p = 0.002, 0.010, 0.038, 0.036, respectively). Low HDL-C (OR=0.869, 95% CI: 0.810–0.933, p < 0.001) and abnormal BP (OR=1.170, 95% CI: 1.090–1.256, p < 0.001) are risk factors for TNs in females.

### Characteristics of the Included Studies

After searching the databases, 63 studies were initially found, and 60 articles were determined to be non-overlapping articles. Thirteen studies were excluded because the literature type was a review and five studies were case reports. Of the remaining 42 studies, 28 studies were excluded because of limited data extraction. The remaining 14 articles were subjected to a full-text evaluation. Finally, 15 studies, including the TIDE research data, were included in the meta-analysis. **Table 2** shows the baseline characteristics, diagnosis criteria of MetS and TN, and quality scores of the 15 included studies, which consisted of 7 case-sectional studies, 4 case-control studies, 3 retrospective studies, and 1 prospective study with a total of 478,981 participants. According to the NOS, one study was rewarded nine stars, seven studies were rewarded eight stars, six studies were rewarded seven stars, and one study was rewarded six stars. The average award of the nine studies was 7.5 stars on a scale of 0–9. All studies were considered adequate for meta-analysis.

**Table 2:** Characteristics and diagnosis criteria of the 15 included studies.

Author	Year	Country	Study Design	Sample size	Gender (M / F)	MetS Diagnosis Criterion					Thyroid Nodule Diagnosis Criterion	Quality assessment
						Abdominal obesity	TG	HDL-C	Blood Pressure	Glucose		
Ayturk	2009	Turkey	case–control	539	——	>102 cm in men, >88 cm in wemen	≥ 1.7 mmol/L (150 mg/dl) or greater	Both men and women < 1.03 mmol/L (40 mg/dl)	≥ 130/85 mmHg	FPG ≥ 6.1 mmol/L (110 mg/dl)	Not clearly stated	7
Chen	2018	China	cross-sectional	9898	4117 / 5781	≥90cm in men, ≥80cm in women or BMI ≥ 30 kg/m2	≥ 1.7 mmol/L or treatment for dyslipidaemia	< 1.03 mmol/L in men or <1.29mmol/L in women or treatment for dyslipidaemia	SBP ≥ 130 or DBP ≥ 85 mmHg or treatment of hypertension	FPG ≥ 5.6 mmol/ L or a history of type 2 diabetes.	thyroid ultrasonography: nodule ≥2mm in diameter.	8
Ding	2017	China	cross-sectional	6365	3070 / 3295	≥ 90 cm in men, ≥ 80 cm in women	≥1.7 mmol/L	< 1.03mmo/L in men, < 1.29 mmol/L in women	SBP ≥ 130 mmHg or DBP ≥ 85 mmHg or have been diagnosed as HBP	FPG ≥ 5.6 mmol/L or have been diagosed as type 2 diabetes	a discrete lesion within the thyroid gland that is radiologically distinct from the surrounding thyroid parenchyma	8
Feng	2017	China	cross-sectional	6495	2427 / 4067	≥90cm in men, ≥80cm in women	≥1.70 mmol/L (150 mg/dL) or specific treatment for this lipid abnormality	<1.03mmol/L (40 mg/dL) in men or <1.29 mmol/L (50 mg/dL) in women or specific treatment for this lipid abnormality	≥130/85mmHg and/or use of anti-hypertensive medications	FPG ≥5.6mmol/L (100 mg/dL) and/or previously diagnosed type 2 diabetes.	Not clearly stated	7
Guo	2019	China	cross-sectional	2606	1338 / 1268	≥90 cm in men, ≥80 cm in women	> 1.7 mmol/L (150 mg/dl) or previous lipid abnormalities, which were described as hypertriglyceridemia	< 1.03 mmol/l (40 mg/dL) in men and < 1.29 mmol/l (50 mg/dL) in women, which were described as low HDL	>130/85 mmHg or previous hypertension diagnosis, which were described as raised blood pressure	>5.6 mmol/l (100 mg/L) or previous type 2 diabetes diagnosis, which were described as dysglycemia.	ultrasound structural focal abnormalities: any area that had a different echogenicity compared to thyroid parenchyma	6
Lai	2020	China	retrospectively	309576	——	BMI ≥25 kg/m2	≥1.7 mmol/L	< 0.9 mmol/ L in male, <1.0 mmol/L in female	SBP≥140 mmHg and/or DBP≥90 mmHg, and/or those who have been confirmed as hypertension and treated	FPG ≥ 6.1 mmol/L and/or PPG ≥7.8 mmol/L	thyroid nodule diagnostic guidelines issued by the American Thyroid Association in 2009	8
Li	2019	China	retrospectively	2068	——	BMI≥25kg/m	≥1.7mmol/L	<0.9mmol/L in men, <1.0 mmol/L in women	≥140/90 mmHg and/or medication	≥ 6.1 mmol/L and/or medication	any nodular lesion that is different from the normal parenchyma of the thyroid gland based on ultrasound	7
Liang	2020	China	prospective	4749	2525 / 2224	≥90 cm in men, ≥80 cm in women	>1.7mmol/L	<1.04mmol/L in men, <1.30mmol/L in female	>130/85mmHg	FPG≥5.6mmol/L	thyroid nodule with diameters equal to or exceeding 2 mm	7
Mayers	2019	Peru	case–control	182	——	≥90 cm in men, ≥80 cm in women	≥1.7mmol/L (150 mg/dL)	< 1.03 mmol/l (40 mg/dL) in men and < 1.29 mmol/l (50 mg/dL) in women	SBP ≥130 mmHg or DBP ≥85 mmHg	≥ 6.1 mmol/L (110 mg/dL)	all lesions of focal increase of volume or consistency located within the thyroid	8
Moon	2018	Korea	cross-sectional	63259	——	≥90cm in men, ≥80cm in women	≥ 1.7 mmol/L (150 mg/dL) or receiving drug therapy for hypertriglyceridemia	< 1.03mmol/L (40 mg/dL) in men or < 1.29 mmol/L (50 mg/dL) in women or receiving drug therapy for reduced HDL-C	≥ 130/85 mmHg or receiving drug therapy for hypertension	FPG ≥ 5.6 mmol/L (100 mg/dL) or receiving drug therapy for hyperglycemia	one or more discrete lesions that were within the thyroid gland but were radiologically distinct from the surrounding thyroid parenchyma	8
Pan	2020	China	cross-sectional	2040	——	BMI ≥ 25	≥ 1.7mmol/L or previous TG abnormalities, described as hypertriglyceridemia	<0.9 mmol/L in men and 1.0 mmol/L in women, described as low HDL-C.	≥ 140/90 mmHg or previously diagnosed with hypertension, described as hypertension	≥ 6.1mmol/L or previously diagnosed with type 2 diabetes, described as hyperglycemia	a lesion within the thyroid gland that has an echoic distinction from the sur- rounding thyroid parenchyma	7
Rendina	2012	Italy	case–control	1422	——	>102 cm in men, >88 cm in wemen	≥1.7 mmol/L (150 mg/dl) or current drug treatment for hypertriglyceridemia	<1.03 mmol/L (<40 mg/dl) in men and <1.3 mmol/L (50 mg/dl) in women or drug treatment for low HDL-cholesterol	SBP ≥130 mmHg, DBP ≥85 mmHg or current antihypertensive drug treatment in a patient with a history of hypertension	FPG ≥5.6 mmol/l (≥100 mg/dl) or drug treatment for elevated blood glucose.	Not clearly stated	7
Shin	2016	Korea	case–control	1990	——	≥ 90 cm in men, ≥ 80 cm in women	≥1.7 mmol/l (150 mg/dl)	<1.03 mmol/L (<40 mg/dl) in men and <1.3 mmol/L (50 mg/dl) in women	≥ 130/85 mmHg or taking antihypertensive medication	FPG ≥5.6 mmol/l (≥100 mg/dl)	discrete lesions distinct from the surrounding thyroid parenchyma, and which had a solid portion regardless of the presence of a cystic portion	8
Su	2019	China	retrospectively	927	365 / 562	≥90cm in men, ≥80cm in women	>1.7 mmol/L, or specific treatment for these lipid abnormalities	<1.03 mmol/L (40 mg/dL) in men and 1.29mmol/L (50mg/dL) in women	SBP >130mmHg and/or DBP >85mmHg	FPG > 5.6 mmol / L	discrete lesions, as they cause distortion of the homogeneous echo pattern of the thyroid gland	8
Our new data (TIDE)	2021	China	cross-sectional	56729	29569 / 27160	≥90 cm in men, ≥85 cm in women	≥1.7 mmol/L	< 1.0 mmol/L in men, <1.3 mmol/L in women	SBP ≥ 130 mmHg or DBP ≥ 85 mmHg or taking antihypertensive medications	FPG ≥ 5.6 mmol/L or PPG ≥ 7.8 mmol/L or HbAIc ≥ 5.7% or self-reported diabetes history or taking hypoglucemia drugs	goiter with nodules exceeding 5mm in diameter	9

WC, waist circumference; TG, hypertriglyceridemia; HDL-C, high density lipoprotein cholesterol; SBP, systolic blood pressure; DBP, diastolic blood pressure; FPG, fasting plasma glucose; PPG, 2h postprandial blood glucose.

### MetS and TNs

**Figure 2:**
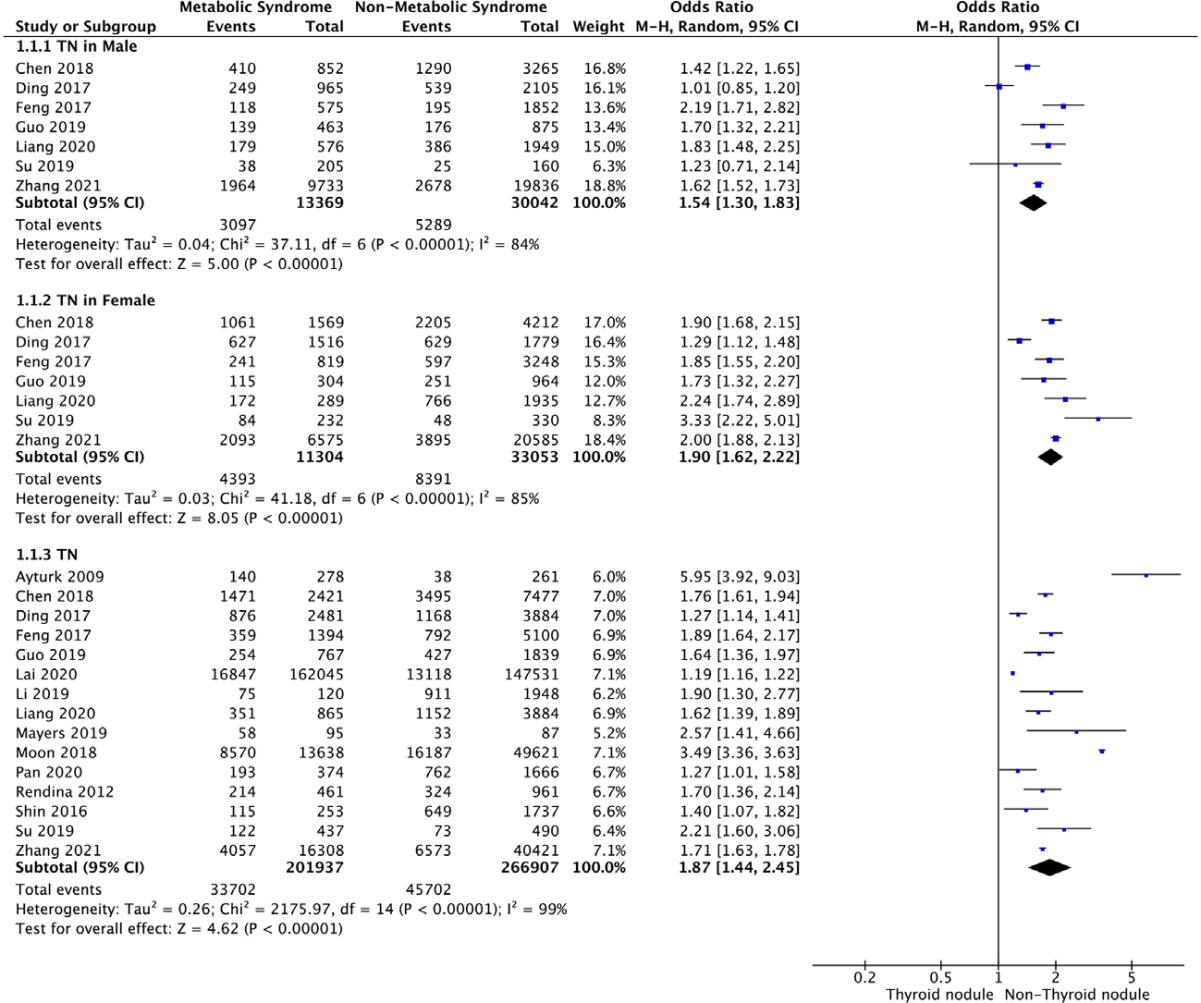
Forest plots of the association between MetS and TNs.

Meta-analysis of the 15 studies that reported relevant data on the relationship between MetS and TNs showed that the combined OR of MetS for TNs in all participants was 1.87 (95% CI: 1.44–2.45, p < 0.001, I^2^ = 99%; **Figure 2**). Subgroup meta-analysis indicated that the presence of MetS increased the risk of TNs in both genders, with a higher OR in females (OR=1.90, 95% CI: 1.62–2.22, p<0.001, I^2 =^ 85%) than males (OR=1.54, 95% CI: 1.30–1.83, p<0.001, I^2 =^ 84%) (**Figure 2**).

### Central Obesity and TNs

**Figure 3:**
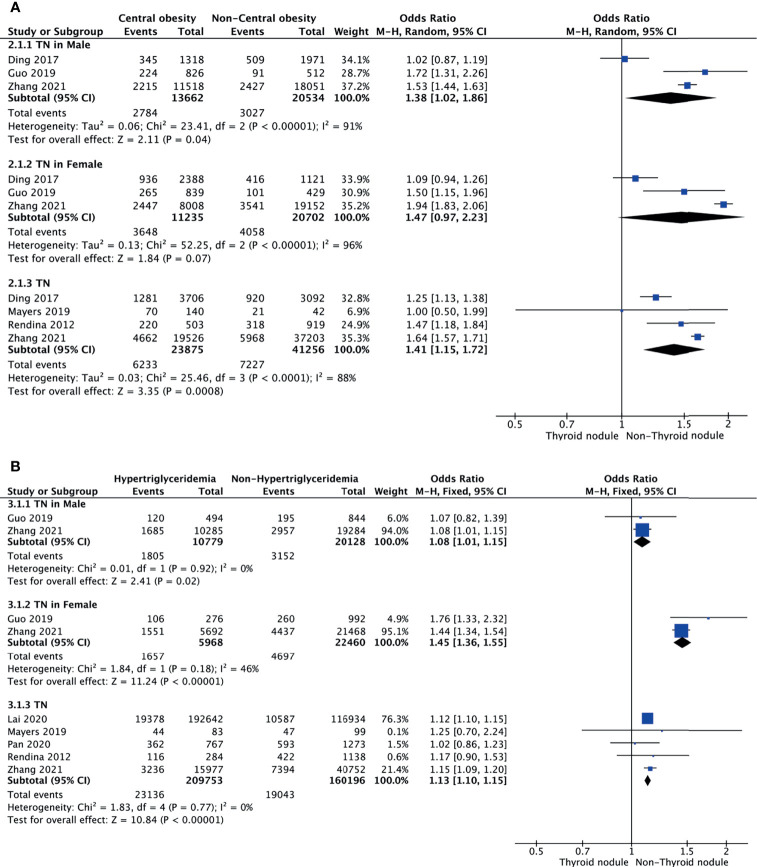
Forest plots of the association among central obesity **(A)**, hypertriglyceridemia **(B)**, and TNs.

Meta-analysis of the four studies that reported relevant data on the association between central obesity and TNs showed that TNs were associated with central obesity (OR = 1.41, 95% CI: 1.15–1.72, p < 0.001, I^2^ = 88%; **Figure 3A**). Subgroup meta-analysis indicated that the presence of central obesity increased the risk of TNs in males (OR = 1.38, 95% CI: 1.02–1.86, p = 0.04, I^2^ = 91%) but not females (OR = 1.47, 95% CI: 0.97–2.23, p = 0.07, I^2^ = 96%) (**Figure 3A**).

### Hypertriglyceridemia and TNs

Meta-analysis of the five studies that reported relevant data on the relationship between hypertriglyceridemia and TNs showed that the combined OR of hypertriglyceridemia for TNs in all participants was 1.13 (95% CI: 1.10–1.15, p < 0.001, I^2^ = 0%; **Figure 3B**). Subgroup meta-analysis indicated that the presence of hypertriglyceridemia increased the risk of TNs in both genders, with a higher OR in females (OR = 1.45, 95% CI: 1.36–1.55, p < 0.001, I^2^ = 46%) than males (OR = 1.08, 95% CI: 1.01–1.15, p = 0.02, I^2^ = 0%) (**Figure 3B**).

### Low HDL-C and TNs

**Figure 4:**
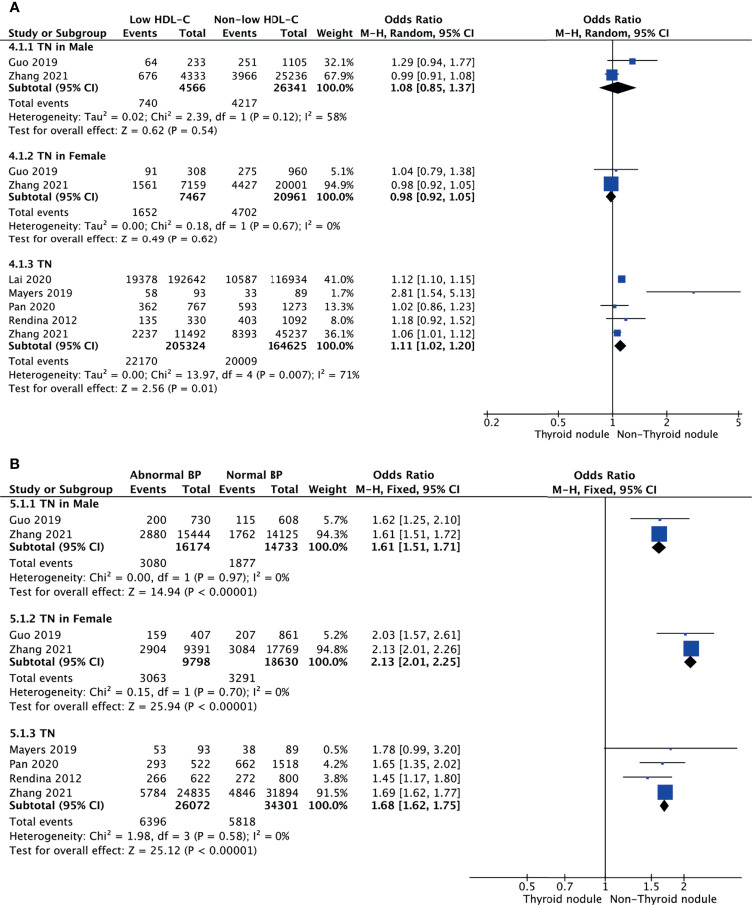
Forest plots of the association among low HDL-C **(A)**, abnormal BP **(B)**, and TNs.

Meta-analysis of the five studies that reported relevant data on the relationship between low HDL-C and TNs showed that the combined OR of low HDL-C for TNs in all participants was 1.11 (95% CI: 1.02–1.20, p = 0.01, I^2^ = 71%; **Figure 4A**). Subgroup meta-analysis indicated that TNs were not associated with low HDL-C regardless of being male (OR = 1.08, 95% CI: 0.85–1.37, p = 0.54, I^2^ = 58%) or female (OR = 0.98, 95% CI: 0.92–1.05, p = 0.62, I^2^ = 0%) (**Figure 4A**).

### Abnormal BP and TNs

Four studies were included in the meta-analysis of the association between abnormal BP and TNs. The results showed that the combined OR of abnormal BP for TNs was 1.68 (95% CI: 1.62–1.75, p < 0.001, I^2^ = 0%; **Figure 4B**). Subgroup meta-analysis indicated that the presence of abnormal BP increased the risk of TNs in both genders, with a higher OR in females (OR = 2.13, 95% CI: 2.01–2.25, p < 0.001, I^2^ = 0%) than males (OR = 1.61, 95% CI: 1.51–1.71, p < 0.001, I^2^ = 0%) (**Figure 4B**). However, it is obvious that TNs were a more important risk factor for abnormal BP in women.

### Hyperglycemia and TNs

**Figure 5:**
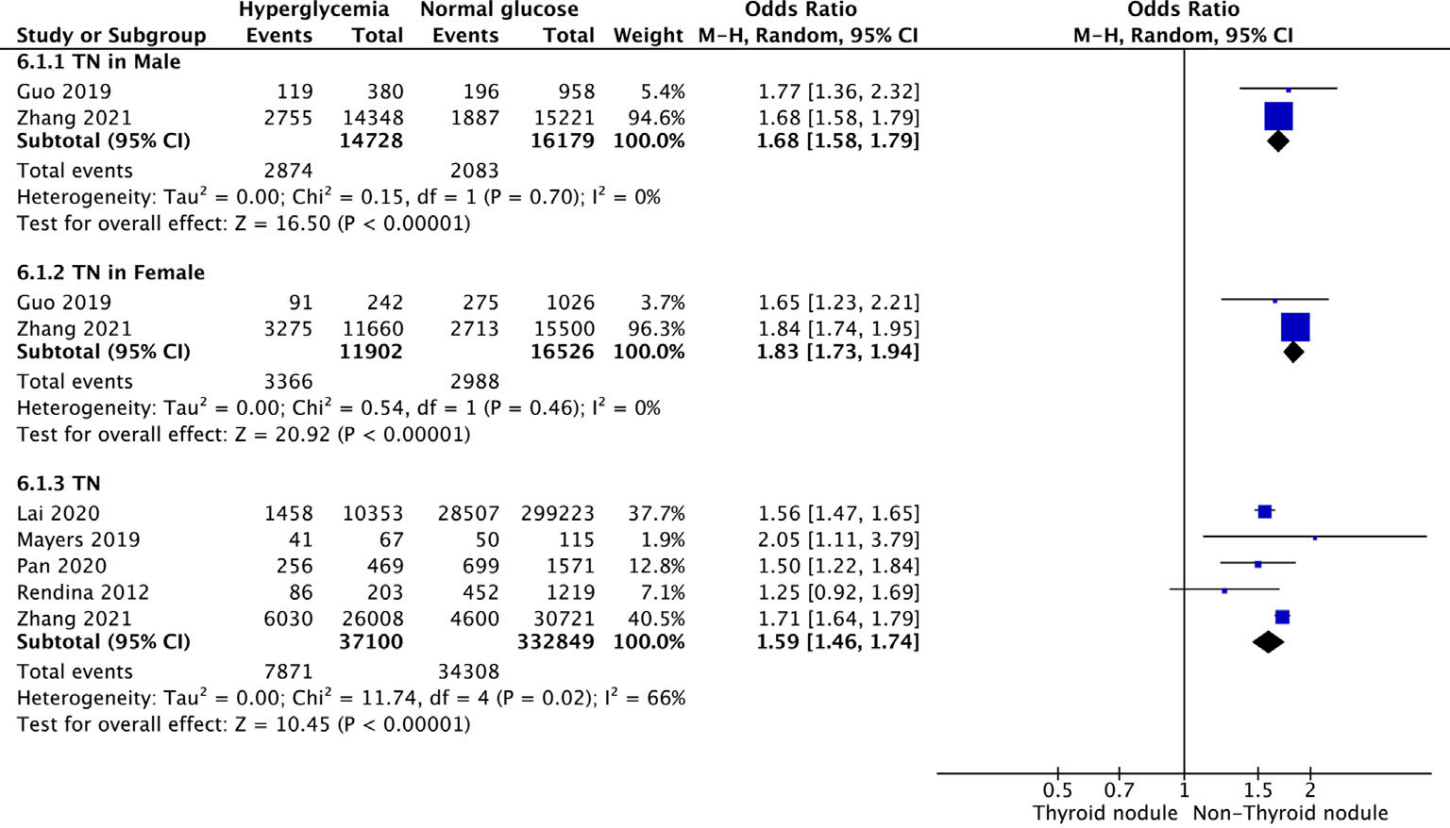
Forest plots of the association between hyperglycemia and TNs.

Five studies were included in the meta-analysis of the association between hyperglycemia and TNs. The results showed that the combined OR of hyperglycemia for TNs was 1.59 (95% CI: 1.46–1.74, p < 0.001, I^2^ = 66%; **Figure 5**). Subgroup meta-analysis indicated that the presence of hyperglycemia increased the risk of TNs in both genders, with a higher OR in females (OR = 1.83, 95% CI: 1.73–1.94, p < 0.001, I^2^ = 0%) than males (OR = 1.68, 95% CI: 1.58–1.79, p < 0.001, I^2^ = 0%) (**Figure 5**).

## Discussion

TNs have become one of the most widespread health issues in the world. With the advancement of imaging equipment, clinicians are finding more TNs, leading to increased prevalence. Several studies in recent years have explored the relationship between TNs and MetS but the conclusions have not been consistent. In order to reduce the error caused by race, sample size, and gender we conducted this meta-analysis to clarify the relationship between TNs and MetS. In addition, a new undisclosed survey result from the China TIDE survey, which involved 56,729 subjects, was included in this meta-analysis. Finally, a total of 468,845 subjects from 15 studies were included in this meta-analysis.

The MetS diagnostic criteria of the TIDE survey used the final report of the National Cholesterol Education Program (NCEP) Expert Panel on Detection, Evaluation, and Treatment of High Blood Cholesterol in Adults (Adult Treatment Panel III, NCEP ATP III) (19) as well as the 2009 Practice Guideline of MetS (1) from the International Diabetes Federation. The results showed that nearly 30% of Chinese people suffer from MetS and 20% suffer from TNs. However, the component diseases of MetS showed a different degree of prevalence. All in all, hyperglycemia and hypertension displayed the highest prevalence, reaching nearly a half. This was followed by central obesity. Although dyslipidemia (included hypertriglyceridemia and low-HDL-C) had the lowest prevalence, it reached nearly one fourth. Such a high prevalence prompts that screening and prevention of MetS and TNs are important. Pan et al. (20) researched an association between hyperhomocysteinemia and TN prevalence in an adult population that included 2040 participants. They observed the prevalence results of MetS and its component diseases were similar to us. The other retrospective study, which included over 300 thousand subjects, reported the detection rate of TNs from healthy people (21). They found the prevalence of hyperlipidemia to be over 60%. This may be because they combined hypertriglyceridemia and low HDL-C, collectively referred to as hyperlipidemia. Compared to females, males displayed a higher prevalence of various metabolic disorders, except for low HDL-C, which was lower than in females. Two cross-sectional studies explored the relationship between MetS and TNs (22, 23). Their results showed that the prevalence of abdominal adiposity or central obesity was higher in females than males, which is different from our results. It might be because their studies had a limited sample size (2606 and 9898, respectively).

Progression of IR can lead to MetS, non-alcoholic fatty liver disease (NAFLD), and type 2 diabetes mellitus (T2DM). The World Health Organization’s (WHO’s) definition of MetS in 1998 is based on the presence of IR with two additional risk factors, including obesity, hypertriglyceridemia, low HDL-C, hypertension, or microalbuminuria, and persons with T2DM were not excluded from the diagnosis (24). The definition of MetS by the American Association of Clinical Endocrinologists (AACE), WHO, and the European Group for the Study of IR (EGIR) focuses on IR detection by an OGTT or hyperinsulinemia-euglycemia clamp. In contrast, the NCEP ATP III defines all simple clinical measures and results of laboratory analyses that do not require proof of IR per se anymore and are easily available to clinicians (25). IR is present in most patients with MetS and is significantly associated with cardiovascular disease (CVD) risk (26). Rezzonico et al. (27) found that IR can increase thyroid volume and is a risk factor for TNs. A case-control study in patients with T2DM found that the volume and size of the TN were positively correlated with HOMA-IR, irrespective of gender, indicating that IR might be a risk factor for TNs (14). In addition, in non-diabetic TN patients, a significant relationship was detected between nodule formation and IR (28). Therefore, the occurrence of TNs may be the result of IR. However, the mechanism behind the relationship between IR and TNs has not yet been researched.

The current obesity epidemic increases the risk of patients developing T2DM. It has also been accompanied by a rise in nodular thyroid disease, mainly in the form of nodular hyperplasia (29). Fussey et al. (30) investigated whether excess adiposity and T2DM are related to TNs. This study used a large number of patients of European descent in the United Kingdom biobank, including 1812 patients who had TNs. They found a positive association between a higher body mass index (OR=1.15) and TNs and between a higher waist-hip ratio (OR=1.16) and TNs. They used mendelian randomization to clarify the causal relationship between central obesity and TNs, and got negative results. However, their research did not perform a subgroup analysis of gender. In this meta-analysis, we found that central obesity was a significant risk factor for TNs, and it was significant in males but not females. Therefore, the real causal relationship between central obesity and TNs could be investigated in different genders using the method of mendelian randomization.

Dyslipidemia is an important component in MetS. Elevated levels of triglycerides are independently associated with an increased risk of CVD (31). Overt or subclinical hypothyroidism negatively affects lipid metabolism. However, it is worth researching whether dyslipidemia displays effects on the thyroid, especially for TNs. Demir et al. (32) assessed the antiproliferative and pleiotropic effects of statins on thyroid volume and nodularity. They found that the total thyroid volume had decreased more in patients receiving 20 mg of rosuvastatin than in the control group (p < 0.05). The maximum nodule size had decreased more in those receiving 10 mg of rosuvastatin (p < 0.05). This research concluded that lipids play a critical role in TN formation. Several researchers have come to different conclusions on whether dyslipidemia can affect the process of TN formation (23, 33–37). The reason for this result may be the interference from different genders. Our meta-analysis found that in different genders, hypertriglyceridemia but not low HDL-C was a risk factor for TNs. This could be because few studies on HDL-C included gender as a subgroup (only 2 studies). Therefore, more literature needs to be included in the meta-analysis to achieve more credible conclusions.

Few studies have researched the relationship between abnormal BP and TNs. Most traceable studies have been based on the relationship between MetS and TNs. However, these studies have not drawn the same conclusions, leading to controversy between abnormal BP and TNs. Some of the large-scale studies, such as Moon et al. (35) and Lai et al. (21), reached a positive conclusion that abnormal BP is a risk factor for TNs. Studies with a small sample size have come to a negative conclusion (34, 37). On the other hand, different diagnosis criteria of abnormal BP might also lead to different conclusions. Three studies defined abnormal BP as SBP≥140 mmHg and DBP≥90 mmHg, while other studies defined it as 130/85 mmHg. In addition, some studies did not include the history of hypertension and the history of taking antihypertensive drugs into the diagnostic criteria. Therefore, in order to draw the most objective conclusion, this meta-analysis included 60,373 subjects to analyze the relationship between abnormal BP and TNs. However, for the different diagnosis criteria, there was no better way to avoid this error in this study.

The predominant consequence of IR is T2DM. IR is thought to precede the development of T2DM by 10 to 15 years. Therefore, the most common manifestation of IR is hyperglycemia (38). Most of the studies concluded that hyperglycemia is a risk factor for TNs (13, 21, 29, 34–37, 39). However, a few studies came to a different conclusion (20, 22, 33). Chen et al. (23) found diabetes to be a risk factor in males but not females. A possible reason for the different conclusions in the above studies is the inconsistent diagnostic criteria for hyperglycemia. Five studies defined hyperglycemia as FPG ≥6.1 mmol/L, while the others’ cut-off value was 5.6 mmol/L. Several studies did not include the history of diabetes nor whether medications were used to treat it. Only two studies included the postprandial plasma glucose, and one study included the HbA1c in the diagnosis criteria of hyperglycemia. Finally, in our meta-analysis, we included 369,949 subjects and found that hyperglycemia was associated with TNs regardless of gender.

A common characteristic in the prevalence of thyroid disorders is in female preponderance. The female-to-male rate ratio is reported at about 3~4:1 for TNs (40). Estrogen receptors are present in thyroid follicular cells in normal and neoplastic tissue (41). *In vitro* studies have demonstrated an estrogen-induced increase in thyroid follicular cell proliferation, suggesting that high estrogen levels could be a risk factor for goiter and TNs (42). In this meta-analysis, the OR of females was higher than males in all analyses, indicating that females with MetS or its components have a higher risk of suffering from TNs than males. Thus, when conducting clinical research on the thyroid, it is recommended that a gender subgroup analysis be conducted to avoid estrogen interference.

An important perspective in recent years is that the escalating global epidemics of T2DM and CVD against the dramatic changes that have occurred in Western and even traditional‐living societies over recent decades with globalization and modernization (43). Several studies have reported that circadian rhythm disturbances may be a major contributor to the contemporary global epidemics of T2DM, CVD and obesity (44). Circadian clocks are highly conserved, endogenous time-keeping mechanisms that are present in virtually all living organisms. These clocks generate self-sustained oscillations with an approximately 24-h period, referred to as ‘circadian rhythms’ (45). Disruptions in circadian clocks have been implicated in various diseases. Generalized disruption of the endocrine system is one of the important mechanisms thought to mediate the adverse effects of circadian misalignment (46). Robust circadian oscillation of clock genes has been observed in primary cultured thyrocytes established from healthy human thyroid tissue and TNs (47). On the other hand, impaired glucose tolerance represents a major systemic effect of circadian disruption (48). Therefore, metabolic diseases may occur through circadian machinery, causing thyroid diseases. Thyroid dysfunction may alter, in a mirror-like manner, circadian clocks, causing metabolic disorders (49). This may provide a new research direction for studying the relationship between TNs and MetS and its components.

As far as we know, this study is the first meta-analysis report to research the relationship between TNs and MetS and its components. It included 468,845 subjects. One of the included studies was our new data from the TIDE survey. In addition, we performed a subgroup analysis of gender. The results unify some previously disputed issues about MetS and its components being risk factors for TNs. However, there are some limitations to this study. Firstly, the diagnosis criteria from all studies were not the same. This included not only the diagnosis criteria of the MetS but also the component diseases. The main differences came from central obesity, abnormal BP, and hyperglycemia. Secondly, since some studies only conducted a population-wide analysis and did not provide data on gender subgroups, we were unable to include enough studies to perform a gender subgroup analysis of MetS and its components. This could have led to a result bias caused by insufficient sample size in the gender subgroup analysis. Finally, most of the included study population was Asian. Therefore, this result may not apply to all populations.

## Conclusion

This meta-analysis demonstrates that TNs are indeed associated with a higher prevalence of MetS. In addition, its component diseases, such as central obesity, hypertriglyceridemia, abnormal blood pressure, and hyperglycemia, are also associated with TNs. Females with MetS or its components have a higher risk of suffering from TNs than males.

## Data Availability Statement

The original contributions presented in the study are included in the article/supplementary material. Further inquiries can be directed to the corresponding authors.

## Ethics Statement

The study was approved by the Ethics Committee of China Medical University. The patients/participants provided their written informed consent to participate in this study.

## Author Contributions

FZ is the first author of this study. WT and ZS are the corresponding author supervising this work. FZ managed the case and drafted the manuscript. YL provided major statistical and technical support. FZ, XY, XW, ZL, BS, and LT assisted in literature review and organizing data from the literature. FZ and CF performed the meta-analysis. WT and ZS reviewed the manuscript. The data of the cross-sectional study is from the TIDE survey group. All authors contributed to the article and approved the submitted version.

## Funding

This study was supported by The Research Fund for Public Welfare, National Health and Family Planning Commission of China (Grant No. 201402005) and The Clinical Research Fund of Chinese Medical Association (Grant No. 15010010589). The funder had no role in study design, data collection or analysis, or in the presentation or publication of the results.

## Conflict of Interest

The authors declare that the research was conducted in the absence of any commercial or financial relationships that could be construed as a potential conflict of interest.

## Publisher’s Note

All claims expressed in this article are solely those of the authors and do not necessarily represent those of their affiliated organizations, or those of the publisher, the editors and the reviewers. Any product that may be evaluated in this article, or claim that may be made by its manufacturer, is not guaranteed or endorsed by the publisher.

## Abbreviations

MetS, Metabolic syndrome; TNs, thyroid nodules; BP, blood pressure; HDL-C, low high-density lipoprotein cholesterol; T2DM, type 2 diabetes mellitus; TIDE, Thyroid Disorders, Iodine Status, and Diabetes Epidemiological; OGTT, oral glucose tolerance test; TG, Hypertriglyceridemia; SBP, systolic blood pressure; DBP, diastolic blood pressure; FPG, fasting plasma glucose ; CI, confidence interval; NOS, Newcastle-Ottawa scale; Ors, odds ratios; NCEP, National Cholesterol Education Program; NAFLD, non-alcoholic fatty liver disease; WHO’s, World Health Organization’s; IR, insulin resistance; AACE, American Association of Clinical Endocrinologists; EGIR, European Group for the Study of IR; CVD, cardiovascular disease.
